# p53R245W Mutation Fuels Cancer Initiation and Metastases in NASH-driven Liver Tumorigenesis

**DOI:** 10.1158/2767-9764.CRC-23-0218

**Published:** 2023-12-29

**Authors:** Denada Dibra, Mihai Gagea, Yuan Qi, Gilda P. Chau, Xiaoping Su, Guillermina Lozano

**Affiliations:** 1 Department of Genetics, The University of Texas MD Anderson Cancer Center, Houston, Texas.; 2 Department of Veterinary Medicine & Surgery, The University of Texas MD Anderson Cancer Center, Houston, Texas.; 3 Department of Bioinformatics and Computational Biology, The University of Texas MD Anderson Cancer Center, Houston, Texas.

## Abstract

Obesity is a significant global health concern. Non-alcoholic fatty liver disease and non-alcoholic steatohepatitis (NASH) are common risk factors for hepatocellular carcinoma (HCC) and are closely associated with metabolic comorbidities, including obesity and diabetes. The *TP53* tumor suppressor is the most frequently mutated gene in liver cancers, with half of these alterations being missense mutations. These mutations produce highly abundant proteins in cancer cells which have both inhibitory effects on wildtype (WT) p53, and gain-of-function (GOF) activities that contribute to tumor progression. A Western diet increases p53 activity in the liver. To elucidate the functional consequences of *Trp53* mutations in a NASH-driven liver tumorigenesis model, we generated somatic mouse models with *Trp5*3 deletion or the missense hotspot mutant p53R245W only in hepatocytes and placed mice on a high-fat, choline-deficient diet. p53R245W in the presence of diet increased fatty liver, compensatory proliferation in the liver parenchyma, and enriched genes of tumor-promoting pathways such as KRAS signaling, MYC, and epithelial–mesenchymal transition when compared with controls in the premalignant liver. Moreover, p53R245W suppressed transcriptional activity of WT p53 in the liver *in vivo* under metabolic challenges, and shortened survival and doubling of HCC incidence as compared with control heterozygous mice. Complete loss of *Trp53* also significantly accelerated liver tumor incidence and lowered time-to-tumor development compared with WT controls. p53R245W GOF properties increased carcinoma initiation, fueled mixed hepatocholangial carcinoma incidence, and tripled metastatic disease. Collectively, our *in vivo* studies indicate that p53R245W has stronger tumor promoting activities than *Trp53* loss in the context of NASH.

**Significance:**

Using somatic NASH-driven mouse models with p53 deletion or mutant p53R245W only in hepatocytes, we discovered that p53R245W increased carcinoma initiation, fueled hepatocholangial carcinoma incidence, and tripled metastases.

## Introduction

According to the World Obesity Atlas 2023, over 4 billion adults and nearly 3 million children will be overweight or obese by 2035, a significant global health concern (1). Hepatocellular carcinoma (HCC) has the fastest rising cancer mortality in the United States and develops primarily in the context of chronic inflammation (2). Non-alcoholic fatty liver disease (NAFLD) is also a common risk factor for HCC and is closely associated with metabolic comorbidities, including obesity and diabetes (2). The incidence of NAFLD, non-alcoholic steatohepatitis (NASH), liver fibrosis, and HCC are expected to continue increasing in the United States. Because of increases in obesity and diabetes, Texas has the highest age-adjusted HCC incidence and mortality rate nationwide (3).

Liver cholangiocarcinoma (CCA) is the second most common liver cancer. Prognosis of these cancers is particularly dismal. Like HCCs, obesity and chronic inflammation are major risk factors for these cancers (4). CCAs were conventionally thought to arise from the biliary duct epithelium; however, recent studies indicate that both hepatocytes and cholangiocytes may give rise to these tumors (5, 6). Such plasticity between cell lineages is particularly more prevalent if the liver is injured (6). Consistent with hepatocyte-to-cholangiocyte reprogramming and plasticity, liver carcinomas can range from HCC to CCA, and intermediates such as hepatocholangial carcinomas (HCC-CCA). These HCC-CCAs have pathologic features of both HCC and CCA, where these features are adjacent or intermixed. Overall survival of patients with these mixed tumors is poor, similar to that of CCAs (7). Intriguingly, genomic sequencing revealed that these mixed tumors were of monoclonal origin, suggesting cellular plasticity/reprogramming accounts for the morphologic heterogeneity of the histopathologic features (7, 8).

*TP53* is the most common altered gene in cancers. This transcription factor is activated in response to stressors such as DNA damage, oxidative stress, inflammation, oncogenic mutations, and metabolic challenges (9). Unlike most tumor suppressors that are usually deleted in cancers, the most frequent alterations in p53 are missense mutations, clustering in particular hotspots in the DNA binding domain, which results in loss of DNA binding. Loss of the other wildtype (WT) allele is also frequent. Furthermore, these missense mutant proteins, unlike WT p53, are highly stable and abundant in cancer cells (10). Such unusual mutational profile and presence of a highly stable protein in the cancer cells led to the hypothesis that these missense mutants have inhibitory effects (IE) on WT p53 and gain-of-function (GOF) properties (9, 11–15). Missense mutants can bind WT p53 and impair its transcriptional activities, termed IE. Xiong and colleagues showed that mice with p53 germline missense mutations (*p53^R248W/+^*, *p53^R270H/+^*, and *p53^R172H/+^*) when treated with low-dose ionizing radiation have lower activation of canonical p53 targets *in vivo* than *p53±* mice (16). In addition, IE of mutant p53 is also prevalent in myeloid malignancies (17). p53 GOF properties are defined as additional functions observed with missense mutants as compared with p53 deletions. For example, studies by Pourebrahim and colleagues found that somatic osteosarcomas expressing the p53R172H mutant doubled the metastatic rate when compared with ones with *p53* loss (18). Similarly, other studies indicate that p53 mutants confer increased metastasis in mouse models and patients (11, 14, 16, 19, 20). Furthermore, genetic studies show that some tumors are dependent on mutant p53 for growth (21, 22). In sum, both inhibitory and GOF are evident *in vivo* under different contexts.

*TP53* is a tumor suppressor and is the most altered gene in HCCs and HCC-CCAs (8, 23). Forty six percent of liver HCCs alter p53, 50% of which are missense mutations. Systemic metabolic challenges, such as a Western diet, stress the liver and fuel liver tumorigenesis (24). In mice, germline mutant p53R172H in concert with immune regulator IL27 receptor deficiency results in spontaneous and sustained liver inflammation, steatosis, and fibrosis (25). With age, these mice develop hepatocyte necrosis, immune cell infiltration, fibrosis, and microsteatosis and macrosteatosis, all phenotypic characteristics of NAFLD and NASH patients. Neither mutant p53, or IL27 receptor deficiency alone results in a liver phenotype (25). These data underscore the strong and cooperative relationship between inflammatory signals and p53 missense mutants particularly in the liver.

The germline models described above do not recapitulate somatic p53 mutations only in liver cells. Given the high prevalence of p53 alterations in liver cancers, it is unknown whether livers with specific p53 alterations respond to metabolic challenges and shape tumor initiation and disease progression. Therefore, we generated a somatic mutant p53R245W model and sought to elucidate the functional consequences of p53 alterations systematically in a NASH-driven liver tumorigenesis model. We discovered that p53R245W suppresses transcriptional activity of WT p53 in the liver *in vivo* under metabolic challenges, and doubles HCC incidence. However, although we observe inhibitory function of p53R45W on p53 WT activity, it is the p53R245W GOF properties that contribute to increased carcinoma initiation, progression, and metastasis.

## Materials and Methods

### Mice

Previously characterized *Trp53^fl/fl^* (26), *Trp53^wm245/+^* (27), *Alb-cre* transgene [a kind donation from Dr. Michele Barton's lab (28)], were bred and crossed to generate *Trp53^fl/fl^*, *Alb-Cre^Tg^* or *Trp53^wm245/fl^*, *Alb-Cre^Tg^*. Cre-negative or Alb-Cre^Tg^ littermate were used as controls. All animals were maintained in the MD Anderson animal facility. All mice were monitored daily. Animals with signs of physical distress were euthanized. All animal studies and procedures were approved by the Institutional Animal Care and Use Committee at MD Anderson Cancer Center. Primer sequence for genotyping is found in Supplementary Table S1. Animals were fed at 3 weeks of age rodent diet with 45 kcal% fat without added choline (D05010402, Research Diets). All animals were euthanized when they had health issues, moribund, or reached 2 years of age. Because of age and poor diet, animals had poor breathing, some had lymphomas (which are common with age), extreme pruritis and skin issues to name a few. Liver of each animal was observed grossly upon dissection and each liver from each animal was paraffin-embedded, processed and hematoxylin and eosin (H&E) were examined by from the pathologist blindly. Liver tumor incidence was annotated if liver tumors were confirmed by the pathologist.

### Histology and Pathology

Tissues harvested from mice were fixed in 10% neutral buffered formalin and paraffin embedded. 4-µm sections were stained with H&E and examined by light microscopy. Tissue processing, paraffin embedding, sectioning, and H&E staining were performed by the MD Anderson Department of Veterinary Medicine and Surgery Histology Laboratory. Tissues were scored blindly by the pathologist. To evaluate liver tumor incidence in the liver at 8.5 months on the diet, livers were sectioned vertically every 5 mm, positioned face down, and H&E slides were evaluated for tumor incidence. The diagnostic criteria are as follows. Oval cell hyperplasia: often originating from the portal areas, entail a single or double row of oval cells between hepatocytes that sometimes appear as duct-like structures. These cells have small oval or round nuclei surrounded by scant pale basophilic cytoplasm and have uniform size and shape. Commonly, there are multiple foci of hyperplastic oval cells throughout the hepatic parenchyma. Bile duct hyperplasia: increased number of small normal bile ducts observed in the portal regions. These bile ducts have well-differentiated epithelial cells, which sometime can be dilated or forming cyst-like structures (cystic biliary hyperplasia). Hepatocellular hyperplasia or hepatocellular hyperplastic nodule: consists of focal areas of hypertrophic and hyperplastic hepatocytes forming a nodular structure but maintaining normal lobular architecture of hepatic parenchyma. The hepatocytes are enlarged and/or increased in number with distinct cytoplasmic morphology and tinctorial pattern. This is a proliferative non-neoplastic change of hepatic parenchyma. Hepatocellular adenoma: is a benign neoplastic lesion of liver consisted of hyperplastic hepatocytes with loss of normal lobular architecture of hepatic parenchyma. In contrast to focal hepatocellular nodules, the hepatocytes form a nodular lesion which is sharply demarcated from surrounding normal liver parenchyma and compresses adjacent normal hepatocytes. Hepatocytes have abnormal cytoplasmic morphology and tinctorial staining pattern and enlarged nuclei. Cholangioma: is a benign neoplastic lesion of bile ducts, which consists of hyperplastic well-differentiated cuboidal epithelial cells forming duct or acini structures. These lesions are well demarcated from the surrounding hepatic parenchyma, and the acini are lined by a uniform layer of cuboidal epithelial cells without invasion into surrounding tissue. Cystic glands could also be observed. The stroma is also sparse in these lesions. Hepatocholangiocellular adenoma: this is a benign neoplastic lesion which has combination of hepatocellular adenoma and cholangioma described above. HCC: this is a malignant neoplasm of liver composed of markedly pleomorphic hepatocytes with invasive growth and loss of hepatic lobular architecture. Neoplastic hepatocytes form either trabecular structures of three or more layer of hepatocytes, acinar structures, adenoid structures of basophilic cuboidal cells, or solid structures of poorly differentiated small, pleomorphic or spindle-shaped hepatocytes. Furthermore, cellular atypia and invasive growth is present. CCA: is a malignant tumor of bile duct epithelium, which unlike cholangioma is characterized by markedly pleomorphic epithelial cells with invasive growth into surrounding hepatic parenchyma, vascular and lymphatic structures. Often, there is moderate to marked amount of hyperplastic fibrous stroma between neoplastic epithelial cells. Hepatocholangiocellular carcinoma: this is a malignant tumor which has features of both HCC and CCA described earlier. The tumor cells are markedly pleomorphic with aggressive invasion into surrounding normal tissues or intravascular metastases.

### RNA Extraction

Flash-frozen tissue was pulverized, and total RNA was prepared using TRIzol Reagent (Invitrogen) and purified using the RNeasy mini kit (Qiagen). Briefly, appropriate 500 µL of TRIzol was added to homogenized tissues and incubated at room temperature for 5 minutes. Chloroform was then added to the tissue/TRIzol mixture and mixed by vortex (chloroform:TRIzol, 1:5 in volume). After incubating at room temperature for 3 minutes, the chloroform/tissue/TRIzol mixture was centrifuged at 12,000 × *g* at 4°C for 30 minutes. The upper phase was transferred to a new tube; 1.5 volumes of 100% ethanol was added to the upper phase, mixed thoroughly by inverting several times, loaded to RNeasy spin column as per manufacture's protocol (Qiagen).

### LOH

LOH was examined by PCR analysis using primers surrounding the mutated site in exon 7 of the Trp53wm-R245W allele, followed by sequencing: 5′-CGGTTCCCTCCCATGCTA-3′, reverse: 5′-AGCGTTGGGCATGTGGTA-3′. The criteria for determining the status of WT *Trp53* alleles are based on the peaks of the mutant and WT allele: (i) if the peaks length is similar between the two for both mutations, then no LOH has occurred, (ii) if only the mutant peak is observed, then complete LOH has occurred, and (iii) if the WT peak is shorter than the mutant, partial LOH as occurred.

### RNA Sequencing

RNA extraction was performed as above. RNA sequencing (RNA-seq) was performed at MD Anderson ATGC with HiSeq4000, generating 76-bp pair end reads. The sample library was prepared using Illumina TruSeq stranded protocol. RNA-seq FASTQ files were processed through FastQC (RRID:SCR_014583), a quality control tool to evaluate the quality of sequencing reads at both the base and read levels. Samples passed FastQC were taken into subsequent analysis. STAR alignment to the GRCm38 was performed with default parameters to generate RNA-seq BAM files (29). Aligned reads were summarized at the gene level using STAR. Gene-level annotation was carried out using the GENCODE (RRID:SCR_014966) annotation, which was downloaded from the GENCODE project (30). The raw count data were processed and normalized by Deseq2 (RRID:SCR_015687) software to identify differentially expressed genes (DEG) between two groups (31). The final *P* value was adjusted using the Benjamini and Hochberg method. A cutoff of gene expression log_2_ fold change of ≥1.0 or ≤−1.0 and an FDR *q*-value of ≤0.05 was applied to select the most significant DEGs. Differential expression analysis was further evaluated using pathway enrichment tools including gene set enrichment analysis (GSEA; ref. 32). MEME Motif Aligment & Search Tool (MAST, RRID:SCR_001783) was used to search promoters (10 kb upstream of starting site) of DEGs (33). MEME-SEA was used to uncover upstream regulators. An E value of 10 was used to score the hits. p53 motif Matrix ID MA0106.3 from Jaspar2022 was used to scan promoters. To generate slopes and *R*^2^ for each gene, normalized read counts averages per group for each gene were calculated. Slopes and *R*^2^ were calculated on these averages from animals with genotypes: LP^+/+^, LP^fl/+^, and LP^245/+^. Genes were further ranked on these criteria: descending slope >1 and determination correlation coefficient *R*^2^ value >0.6.

### IHC and Immunofluorescence

IHC was performed using standard methods. Endogenous biotin in the liver was blocked via incubation of slides for 15 minutes with 0.001% avidin in PBS follow by 15 minutes incubation with 0.001% biotin in PBS, and tissue sections were stained with antibodies against CK19 (1:5,000, Ab133496, Abcam, RRID:AB_11155282), TRIS-EDTA pH 9.0 was used as antigen retrieval. Visualization was performed using avidin–biotin complex and DAB (3,3′-diaminobenzidine) kits (Vector Laboratories) and slides were counterstained with hematoxylin. CSP1 (1:200, Ab45956, Abcam, RRID:AB_941153, Citrate buffer pH 6.0 as antigen retrieval).

Immunofluorescence staining was performed as previously described. Paraffin-embedded tumor sections were deparaffinized and rehydrated, and antigen retrieval was performed in TRIS-EDTA pH 9.0 or Citrate buffer pH 6.0. Slides were blocked in PBS containing 3% fish gelatin (VWR) for 20 minutes. Tissue sections were incubated with anti-rabbit cleaved caspase 3 (CC3; 1:200, 9661, Cell Signaling Technology, RRID:AB_2341188, Citrate buffer pH 6.0), anti-rabbit Ki67 (1:800, Ab15580, Abcam, RRID:AB_443209, Citrate buffer pH 6.0), anti-rat, CK19 (1:50, Troma III, Developmental Studies Hybridoma Bank, TRIS-EDTA pH 9.0) overnight at 4°C, followed by an anti-p53 antibody (CM5, Leica Biosystems; 1:100) overnight at 4°C and then incubated with a secondary antibody labeled with AlexaFluor 555 and AlexaFluor 488 (Thermo Fisher Scientific; 1:600 RRID:AB_162543, RRID:AB_2633275). The sections were counterstained with DAPI (Thermo Fisher Scientific). Images were acquired using a Nikon 80i upright widefield fluorescence microscope with NIS-Elements imaging software.

### Picrosirius Red Staining

Deparaffinized sections were stained in Wiegert's Hematoxylin Kit (Solutions A & B, catalog no. 25373, Polysciences, Inc.) for 8 minutes, washed with tap water until clear (two to four times). Next, sections were stained in PicroSirius red Direct Red 80 (catalog no. 365548-5G, Sigma-Aldrich) 0.5g, in Picric Acid, Saturated (catalog no. LCS18670-1, LabChem) 500 mL for 1 hour, followed by tap water wash until clear (four to five times). Finally, sections were dipped 5 minutes in acid wash with tap water and glacial acetic acid 5 mL/Liter, two times.

### Red Oil O Staining

Red Oil O staining was performed by MD Anderson's Department of Veterinary Medicine and Surgery's histology laboratory using Red Oil O staining kit (ORK-1-IFU ScyTek Laboratories) kit according to the manufacturer's instructions.

### qRT-PCR

RNA was purified as described above. cDNA was synthesized using the iScript reverse transcription supermix kit (Bio-Rad). Quantitative PCR was performed with CFX96 (Bio-Rad), and data were analyzed using CFX Maestro Software (Bio-Rad). mRNA levels were calculated using the ΔCt method and normalized to those of large ribosomal protein (*RPLP0*). Results are expressed as fold changes relative to the controls. The qRT-PCR primer sequences are listed in Supplementary Table S1.

### Statistical Analysis

All data are represented as mean ± SEM. Graphpad 9.0 Prism (RRID:SCR_000306) was used to perform all statistical analyses. Significance of differences between groups was evaluated by Student *t* test (two groups) or ANOVA. *P* < 0.05 was considered significant, or Fisher exact test. Comparison of survival curves was done using a log-rank Mantel–Cox test.

### Data Availability Statement

RNA-seq data reported in this article have been deposited in the Gene Expression Omnibus database, GSE230057.

## Results

### p53R245W Induces Fatty Liver and Compensatory Proliferation in Hepatocytes *in vivo* under Metabolic Challenges

The most common p53 hotspots in liver cancers occur at amino acid R249, followed by R248 (34, 35). p53R249 is a frequent missense mutation due to aflatoxin B1, which is particularly common in Southern Asia and Sub-Saharan Africa (36). The p53R248 (corresponding to murine p53R245) is one of the most common missense alterations in HCCs. Given the rise of HCC incidence in the Western world associated with obesity, we sought to understand how p53R245W or p53 loss cooperate with metabolic challenges to drive liver tumorigenesis.

**FIGURE 1:**
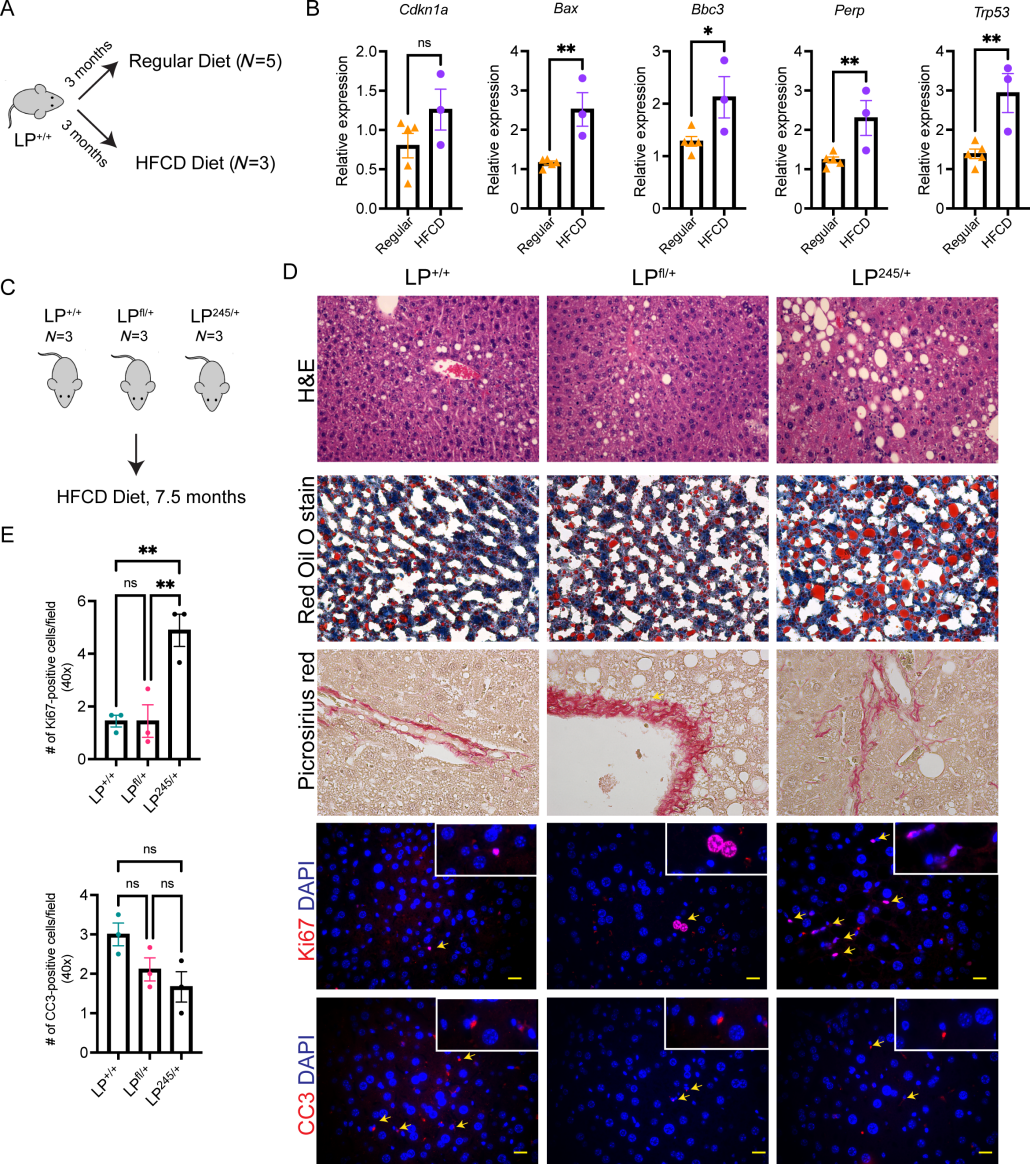
p53R245W induces fatty liver and compensatory proliferation in hepatocytes *in vivo* under metabolic challenges. **A,** Schematic of liver samples used. **B,** Expression of p53 target genes in the liver of animals fed regular chow (*N* = 5) or HFCD diet (*N* = 3) for 3 months. Each dot represents an individual animal. **C,** Schematic of animals with various genotypes fed a HFCD diet for 7.5 months. **D,** Representative H&E, Red Oil O stain, Picrosirius red, Ki67, and CC3 in liver of animals from LP^+/+^ (*N* = 3), LP^fl/+^ (*N* = 3), and LP^245/+^ (*N* = 3). Yellow scale, 20 µm. **E,** Quantification of Ki67-, and CC3-positive cells in livers of mice with the indicated genotypes fed for 7.5 months on a HFCD diet. Each dot represents an individual animal. *, *P* < 0.05; **, *P* < 0.002; ns, non-significant. Figure panels A, C were created in BioRender.com.

Western diet stresses the liver and is associated with NAFLD and NASH. We examined whether a Western diet increases p53 activity in the liver. To mimic a Western diet and fuel liver tumorigenesis, mice were placed on high-fat, choline-deficient (HFCD) diet starting at week 3 postnatally (24) for 3 months (Fig. 1A). Well-known transcriptional p53 targets such as *Bax*, *Bbc3* (*Puma*), *Perp* were significantly elevated in the livers of animals fed a HFCD diet (Fig. 1B) when compared with those fed regular chow. *Trp53* levels were also elevated. *Cdkn1a* (*p21*) levels were also elevated but did not reach statistical significance. These data indicate that a Western diet elevate p53 transcriptional activity in the liver.

Next, to examine the cooperation of p53 alterations and diet to drive liver cancer formation, we generated three cohorts: one cohort deletes one allele of *Trp53* specifically in hepatocytes (*p53^fl/+^*, *Alb-Cre^Tg^*; labeled LP^fl/+^ for liver specific *Trp53* deletion), one has a somatic p53 missense mutation R245W specifically in hepatocytes (*p53^wm245/+^*, *Alb-Cre^Tg^*; LP^245/+^), and the third has no alterations (*p53^+/+^*, *Alb-Cre^Tg^*; LP^+/+^). The *p53^wm245^*allele expresses WT p53, which is converted to p53R245W in a Cre-mediated manner (27). The Alb-Cre transgene recombines alleles in both hepatocytes and cholangiocytes during embryogenesis, leaving other cells WT for p53 (37). While p53 activity was elevated after 3 months in the diet, no phenotypic differences between genotypes were observed via H&E, at this timepoint (Supplementary Fig. S1A). Next, we aged LP^+/+^, LP^fl/+^, and LP^245/+^ animals for 7.5 months on the HFCD diet and examined their livers via H&E (Fig. 1C). p53R245W accelerates fatty liver, as indicated by H&E and Red Oil O staining (Fig. 1D). While loss of one *Trp53* allele accelerates collagen deposition as indicated by Picrosirius red staining when compared with WT p53, p53R245W accelerates collagen deposition not only around the vessels, but also in the liver parenchyma (Fig. 1D). Recurrent and chronic stressors induce a cycle of cell death followed by compensatory proliferation mechanisms, the latter when left unchecked, drive tumorigenesis. We therefore examined CC3 (a marker of apoptosis) and Ki67 (a marker of proliferation). While the number of CC3-positive cells were reduced in LP^fl/+^ and LP^245/+^ livers when compared with control animals, no statistical significance was reached (Fig. 1D and E). Meanwhile, the number of Ki67-positive cells significantly increased in LP^245/+^ livers when compared with control animals (Fig. 1D and E); however, loss of one *Trp53* allele did not affect the number of proliferating cells, implicating p53R245W in liver tumorigenesis.

### p53R245W Suppresses Transcriptional Activity of WT p53 in the Liver *in vivo* under Metabolic Challenges

**FIGURE 2:**
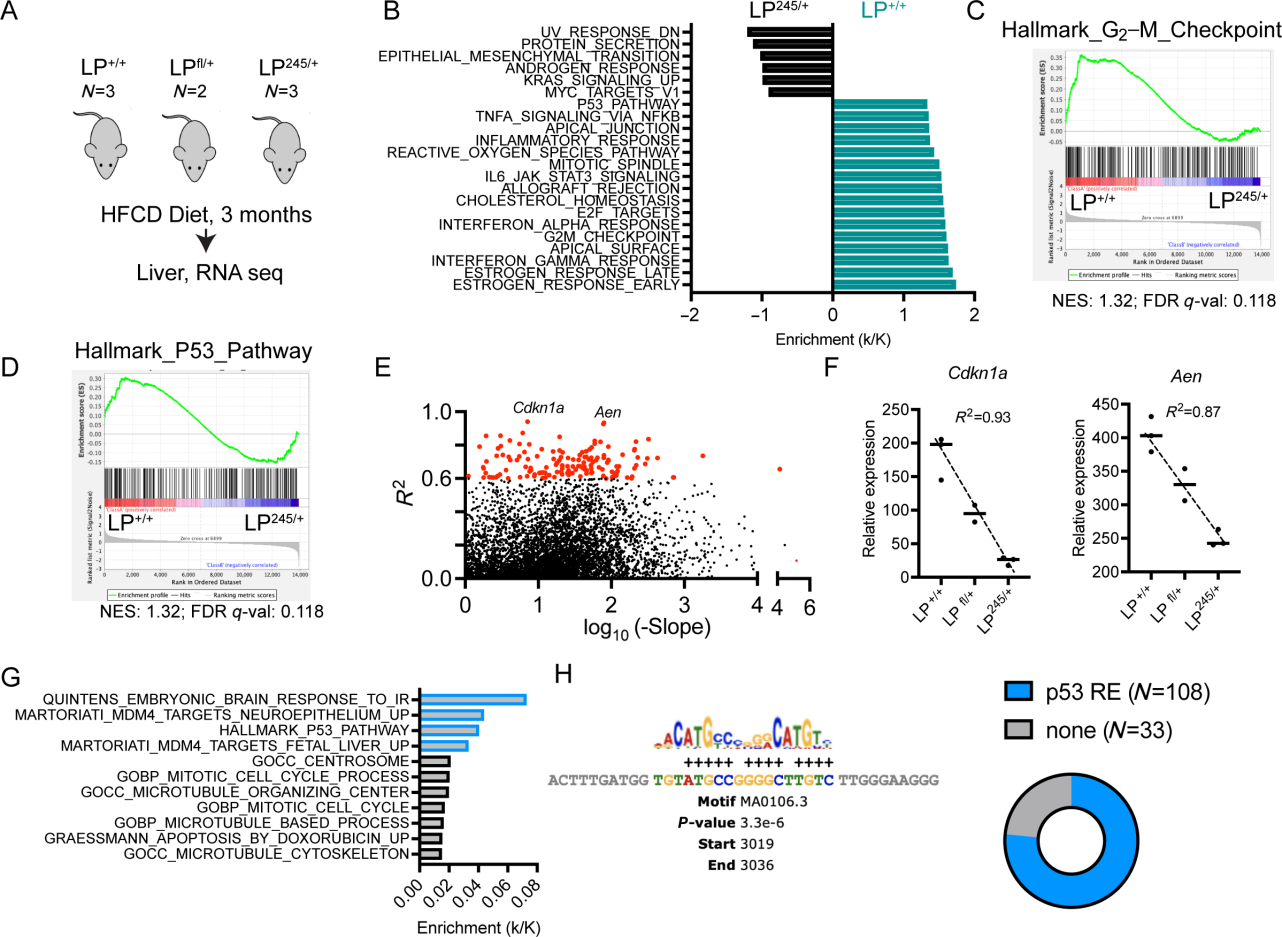
p53R245W suppresses transcriptional activity of WT p53 in the liver *in vivo* under metabolic challenges. **A,** Schematic of liver samples submitted for bulk RNA-seq (LP^+/+^, *N* = 3; LP^fl/+^, *N* = 2; and LP^245/+^, *N* = 3). **B,** GSEA pathway analysis from dataset Hallmark (ranked by NES, k/K) enriched in livers from LP^+/+^ or LP^245/+^ genotypes. NES, normalized enrichment score. **C** and **D,** GSEA enrichment plots indicating that p53R245W inhibits the G_2_–M checkpoint and p53 pathway. NES, normalized enrichment score; FDR, false discovery rate *q*-value. **E,** Expression levels of inhibitory (IE) genes based on negative slope and *R*^2^. Genes with *R*^2^ > than 0.6, (141) are marked in red. **F,** Normalized read counts for the top two genes based on *R*^2^, *Cdkn1a* and *Aen*, from the inhibitory analysis. **G,** GSEA pathway analysis performed on inhibitory genes identified in analysis in B. **H,** p53 motif and proportion of IE genes with p53 response element regulated by p53 using MEME MAST (motif alignment and search tool). Figure panel A was created in BioRender.com.

p53R245W in combination with a HFCD diet induces fatty liver and cellular proliferation when compared with p53 WT animals. To evaluate the physiologic consequences of p53 alterations in hepatocytes, age-matched males from LP^+/+^, LP^fl/+^, and LP^245/+^ were placed on the HFCD diet for 3 months (early timepoint), and livers from these animals were subjected to RNA-seq (Fig. 2A). GSEA of premalignant livers from LP^245/+^ compared with LP^+/+^ revealed that tumor-promoting pathways such as KRAS signaling, MYC, and epithelial–mesenchymal transition (EMT) pathways were enriched in LP^245/+^ (Fig. 2B). Meanwhile, immune-related pathways, cholesterol homeostasis, G_2_–M checkpoint, and p53 pathways were enriched in LP^+/+^ livers when compared with LP^245/+^ ones (Fig. 2B–D).

An IE of mutant p53 on WT p53 is observed in spleen and thymus of heterozygous germline mutant p53 animals (16). Given that p53 and G_2_–M checkpoint (regulated by p53) pathways were enriched in LP^+/+^ livers, we next investigated whether p53R245W exerts an IE over the WT allele *in vivo.* Genes activated by WT p53 would be expressed highest in LP^+/+^ mice, and progressively decrease in LP^fl/+^ and LP^245/+^ mice. While the p53 transcriptome after a stressor like irradiation is well understood, downstream p53 targets in the liver challenged by diet are not characterized. To identify an IE transcriptome in an unbiased manner of the p53R245W in the metabolically challenged livers, genes from LP^+/+^, LP^fl/+^, and LP^245/+^ were ranked on the basis of descending slope and correlation coefficient *R*^2^. Genes were further ranked on these criteria: descending slope >1 and determination correlation coefficient *R*^2^ value >0.6. 141 genes satisfied both requirements (Fig. 2E). *Cdkn1a* (p21) and *Aen*, both p53 targets, were in the top genes from this analysis, with p21 being one of the most robust p53 targets (Fig. 2F; Supplementary Table S2). The fact that *Cdkn1a* scored as second highest gene in this analysis underscores confidence in the analysis, and on the inhibitory functions of p53R245W in this model. Next, GSEA on these inhibitory genes revealed that top four pathways were p53 related (Fig. 2G). Subsequently, we performed MEME MAST (motif alignment and search tool) analysis of the promoters from our inhibitory analysis (141 genes) and found that 77% (108 genes) of these IE genes have a p53 response element 10 kb upstream of their promoters, (Fig. 2H; Supplementary Table S3). To exclude the possibility that p53 dependency is by random chance, 141 random genes were examined for a p53 response element 10 kb upstream of their promoters. Only 5/141 (3.5%) genes had a p53 response element 10 kb upstream of their promoter (Supplementary Fig. S1B), minor when compared with 77% identified in Fig. 2H. Furthermore, we also performed an unbiased motif analysis of the promoters of these to gain an understanding of what may be driving their expression and found ZNF460 as the top regulator of these genes (using MEME-SEA; Supplementary Fig. S1C). Alternatively, some of these genes could also be indirect targets of p53. In sum, our data not only indicate that p53R245W has inhibitory functions in the liver *in vivo* under metabolic stress, but we also have identified in an unbiased manner an IE transcriptome of p53R245W specific to the liver challenged by diet.

### p53R245W Displays GOF Activities to Accelerate Liver Tumorigenesis

**FIGURE 3:**
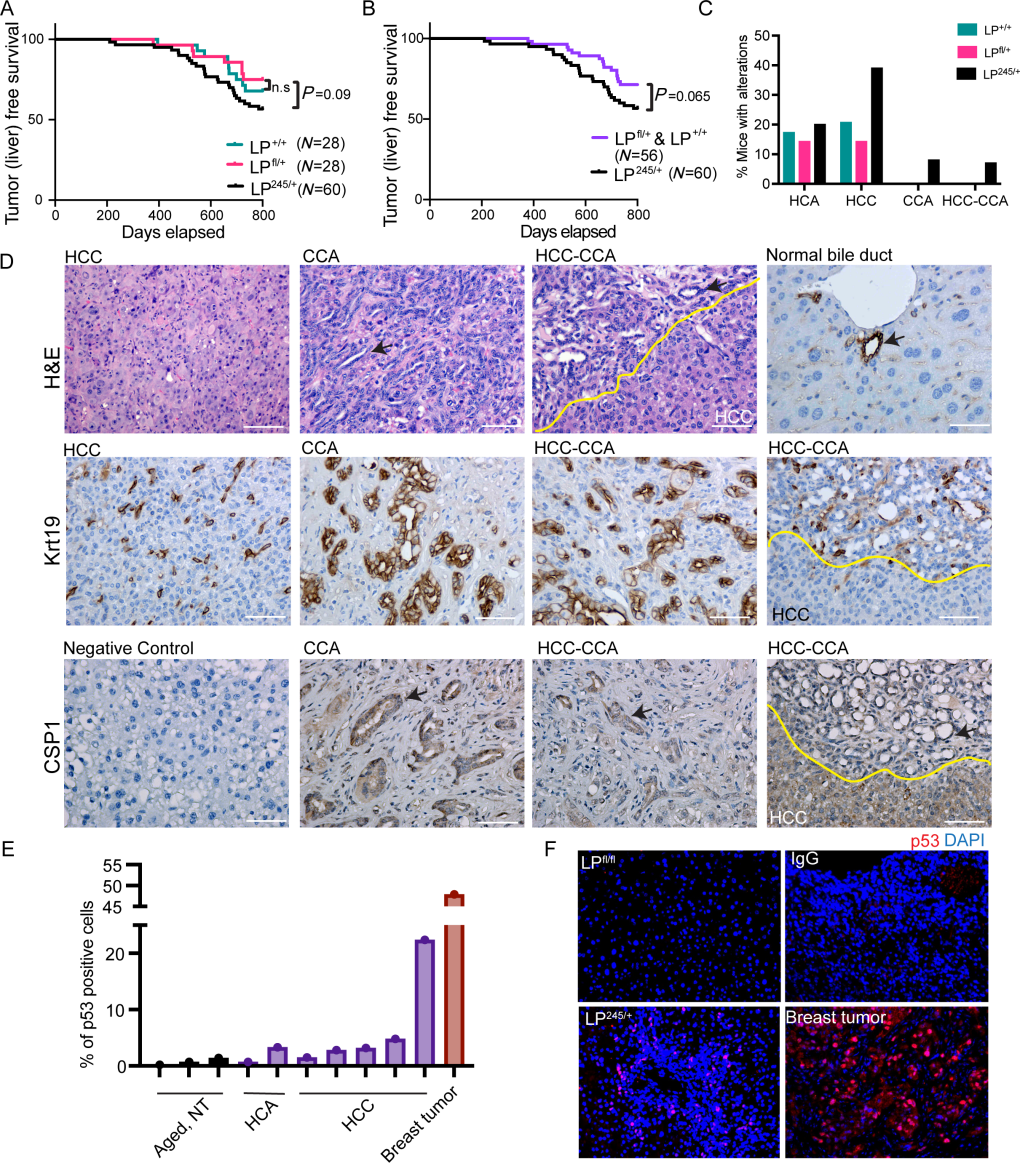
p53R245W displays GOF activities in the liver during tumorigenesis. **A,** Kaplan–Meier survival curves for animals with genotypes LP^+/+^ (*N* = 28), LP^fl/+^ (*N* = 28), and LP^245/+^ (*N* = 60), with hepatocellular adenomas and carcinomas. **B,** Kaplan–Meier survival curves for LP^+/+^ (*N* = 28) and LP^fl/+^ (*N* = 28) combined versus LP^245/+^ (*N* = 60), with hepatocellular adenomas and carcinomas. **C,** Pathologic subtypes of tumors from LP^+/+^ (*N* = 28), LP^fl/+^ (*N* = 28), and LP^245/+^ with hepatocellular adenoma (HCA), HCC, CCA, and HCC-CCA. **D,** Representative H&E sections of liver tumors from mice with different pathologic subtypes. Keratin 19 staining of bile duct, CCA, HCC-CCA. CSP1 stain of CCA and HCC-CCA. Negative control, absence of primary antibody. Arrows point to bile ducts which are positive for CSP1 staining. **E,** Quantification of p53 stability in liver tumors from LP^245/+^ genotype. We used a representative breast tumor as a positive control and aged livers without tumors as negative controls. **F,** Immunofluorescence imaging of mutant p53 (red) and DAPI (blue) in liver tumors.

Transcriptome data described above indicated that p53R245W enhances tumor-promoting pathways such as KRAS signaling, MYC, and EMT pathways in the premalignant liver (Fig. 2B). We therefore assessed whether p53R245W accelerated liver tumorigenesis. Animals from LP^+/+^ (*N* = 28), LP^fl/+^ (*N* = 28), and LP^245/+^ (*N* = 60) were placed on the HFCD diet at 3 weeks of age and were monitored over time. Comparison of LP^fl/+^ to LP^+/+^ animals showed similar survival; 20% of mice have liver cancers by 800 days, indicating that one WT *Trp53* allele is sufficient in inhibition of liver tumorigenesis (Fig. 3A). However, LP^245/+^ animals had a worse survival when compared with LP^fl/+^, indicating that p53245W had additional functions when compared with *Trp53* loss alone. Because LP^fl/+^ and LP^+/+^ mice had similar survival curves, we combined and compared them with LP^245/+^ mice; LP^245/+^ mice showed worse survival with increased *P* value (Fig. 3B). A small cohort of LP^+/+^ (*N* = 18), LP^fl/+^ (*N* = 7), and LP^245/fl^ (*N* = 10) fed regular chow were monitored over time. No significant differences in survival or tumor incidence were observed (Supplementary Fig. S2A), implicating diet as a stressor is critical for the observed effects of p53R245W (Fig. 3A and B). Given the higher incidence of liver tumors in males than females both in humans and mice, similar analyses were also performed based on gender. LP^245/+^ showed decreased survival from both genders (Supplementary Fig. S2B and S2C).

To examine the potential cooperative role of LOH for *Trp53*, we performed Sanger sequencing on 19 tumors from LP^245/+^ mice. The majority (68%) retained the WT allele (showed no LOH), some showed partial LOH, ranging in loss of WT allele from 15% (*N* = 1), 25% (1), 33% (*N* = 2), and 50% (*N* = 1). Only one tumor showed complete LOH (Supplementary Fig. S3). These data indicated marginal pressure to delete the *Trp p53* WT allele.

Liver carcinomas are of several types: HCC, CCA, and intermediates such as HCC-CCAs. Next, we compared pathologically the incidence of different kinds of liver tumors among the different genotypes. Examination of these tumors from both genders revealed that all three genotypes had similar incidence of hepatocellular adenomas (ranging from 17%–20%; Fig. 3C). However, LP^245/+^ had double the incidence of HCC when compared with LP^fl/+^ (38% vs. 14%) with 20% of LP^+/+^ mice developing HCC (Fig. 3C). While histopathologic examination identified all tumors from LP^fl/+^ and LP^+/+^ as hepatocellular adenomas and HCC, tumors from LP^245/+^ were more diverse, as CCA and HCC-CCAs were also observed. HCC-CCA is a rare tumor presenting the histologic characteristics of both HCC and intrahepatic CCA. These data indicated a greater tumor plasticity in the LP^245/+^ cohort (Fig. 3C and D), a phenotype not observed in the other two cohorts, indicating GOF properties. Bile ducts and CCA cancers are positive for epithelial cell marker keratin 19. Evaluation of keratin 19 in CCA and HCC-CCAs of LP^245/+^ confirmed biliary lesions within these CCA and HCC-CCA (Fig. 3D). In addition, in some HCCs from LP^245/+^ mice, single-nuclei keratin 19-positive cells rather than duct-like structures were observed, perhaps reflecting transdifferentiating hepatocytes into biliary cells (Fig. 3D). Next, we stained these tumors for carbamoyl phosphate synthetase 1 (CSP1), a marker of cells of hepatocellular origin (38). While the HCC areas stained positively for CSP1, both biliary lesions in these cancers stained positively, indicating that these lesions express markers of both biliary and hepatocellular cells, implicating transdifferentiation ability in these cells (Fig. 3D).

p53 GOF properties are defined as additional functions observed with missense mutants as compared with *p53* deletions. We observed CCA and HCC-CCAs in LP^245/+^ cohort, but not in LP^fl/+^ animals, indicating GOF properties. p53 stability of missense mutants is associated with GOF (10, 21, 39). Therefore, we stained seven tumors, five HCC and two hepatocellular adenoma (HCA) for p53 and quantified the number of cells with a stable mutant p53. In addition, we also stained three aged livers that did not develop tumors and one breast tumor generated from mice expressing p53R245W in breast epithelium. These breast tumors have highly stable mutant p53 that shows GOF. Most HCC from LP^245/+^ showed very low p53 stability, ranging from 0%–5% of total cells with a stable p53 (Fig. 3E and F). Only one of five HCC had approximately 20% stable p53; none of the HCA had stable p53. As a positive control, more than 50% of breast cancer cells were positive for mutant p53. Overall, these data indicate that p53R245W GOF properties in the liver may be independent of protein stability.

### p53R245W GOF Properties in Absence of WT p53 Increase Incidence of Carcinomas and Mixed HCC-CCA Cancers

**FIGURE 4:**
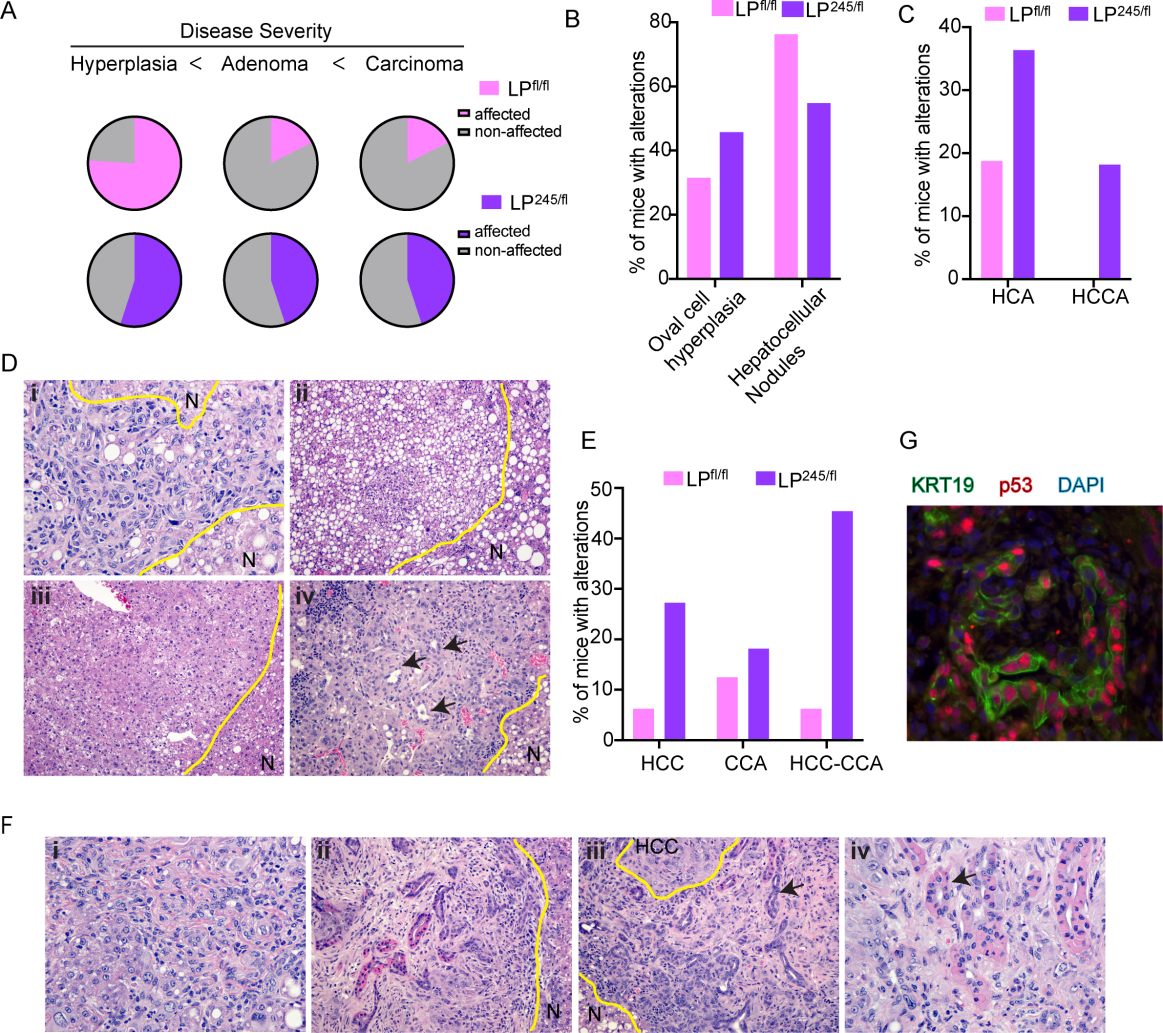
p53R245W GOF properties increase carcinoma incidence and mixed HCC-CCA cancers. **A,** Quantification of animals from LP^fl/fl^ (*N* = 17), LP^245/fl^ (*N* = 11) with hyperplasia (left), adenoma (middle), and carcinomas (right) after 8.5 months on the diet. Quantification of animals from LP^fl/fl^ (*N* = 17), LP^245/fl^ (*N* = 11) with subtypes of hyperplasia (**B**) or adenomas (**C**) after 8.5 months on HFCD diet. HCA, hepatocellular adenoma: HCCA, hepatocholangial adenoma. **D,** Representative H&E-stained sections of liver tumors from mice with different pathologic subtypes such as oval cell hyperplasia (i), hepatocellular hyperplastic nodule (ii), hepatocellular adenoma (iii), and hepatocholangial adenoma (iv). Arrows point at bile ducts. **E,** Quantification of pathologic subtypes of liver carcinomas from LP^fl/fl^ (*N* = 17), LP^245/fl^ (*N* = 11) at 8.5 months on the diet. HCC, hepatocellular carcinoma; CCA, cholangiocarcinoma, HCC-CCA, hepatocholangial carcinomas. **F,** Representative H&E-stained sections of liver tumors from mice with different pathologic subtypes: hepatocellular carcinoma (i), cholangial carcinoma (ii), HCC-CCA (iii), and zoomed in HCC-CCA (iv). **G,** Immunofluorescence imaging of mutant p53 (red), KRT19 (green) and DAPI (blue) in liver tumors in a HCC-CCA tumor from LP^245/fl^ cohort. N**,** normal tissue.

To further distinguish inhibitory from GOF properties of mutant p53R245W *in vivo*, we generated two additional cohorts, LP^245/fl^, and LP^fl/fl^. To assess tumor initiation in this model in an unbiased manner, we aged LP^fl/fl^ (*N* = 17) and LP^wm245/fl^ (*N* = 11) male animals for 8.5 months on the HFCD diet. Males develop liver malignancies at a higher rate than females (40). In a pilot study (6 mice), we observed early signs of tumorigenesis in at least 50% of the males after 8.5 months on the diet. To comprehensively annotate liver tumor initiation, liver lobules were sectioned serially and scored for abnormalities by a pathologist. We observed an increase in incidence of hyperplasia in LP^fl/fl^ mice where 76% of animals were affected versus 55% of LP^245/fl^ mice (Fig. 4A). However, only 17.6% of LP^fl/fl^ animals progressed to adenomas, while 45% of LP^245/fl^ also had adenomas (∼3-fold increase; Fig. 4A). Finally, only 17.6% of LP^fl/fl^ animals progressed to malignant carcinomas, while 45% of LP^245/fl^ had carcinomas (∼3-fold increase; Fig. 4A). These data indicate that p53R245W has GOF properties that increase disease progression and carcinoma incidence.

Next, we assessed more specifically the type of hyperplasias (which can be oval cell, bile duct or hepatocellular hyperplasia), adenomas (hepatocellular, cholangial, or hepatocholangial), and carcinomas (HCC, CCA, or HCC-CCA) observed. LP^245/fl^ animals had an increase in oval cell hyperplasia (45% vs. 31%), and a decreased in incidence of hepatocellular hyperplasia (54% vs. 76%) compared with LP^fl/fl^ ones (Fig. 4B). However, more cells from LP^wm245/fl^ mice progressed to adenoma with a 20% of animals displaying metaplastic hepatocholangial adenomas, a subtype not seen in LP^fl/fl^ (Fig. 4C and D). Next, we compared the incidence of carcinomas based on subtype. While the incidence of CCA was similar between the two genotypes, we observed a higher incidence (4.3-fold; Fig. 4E and F) of HCC and mixed HCC-CCAs (7.3-fold increase; Fig. 4E and F). Given that p53R245W has GOF properties that increase cancer cell plasticity, we tested whether mutant p53 was stable in the CCA portion of HCC-CCA tumor with a p53 missense mutation. Indeed, immunofluorescent staining revealed costaining for keratin 19/mutant p53-positive cells in HCC-CCAs (Fig. 4G). Collectively, these data indicate that p53R245W has GOF properties that increase both carcinoma incidence and that of mixed cancers such as HCC-CCA.

### p53R245W GOF Mechanisms Triple Metastatic Incidence in Liver Tumors

**FIGURE 5:**
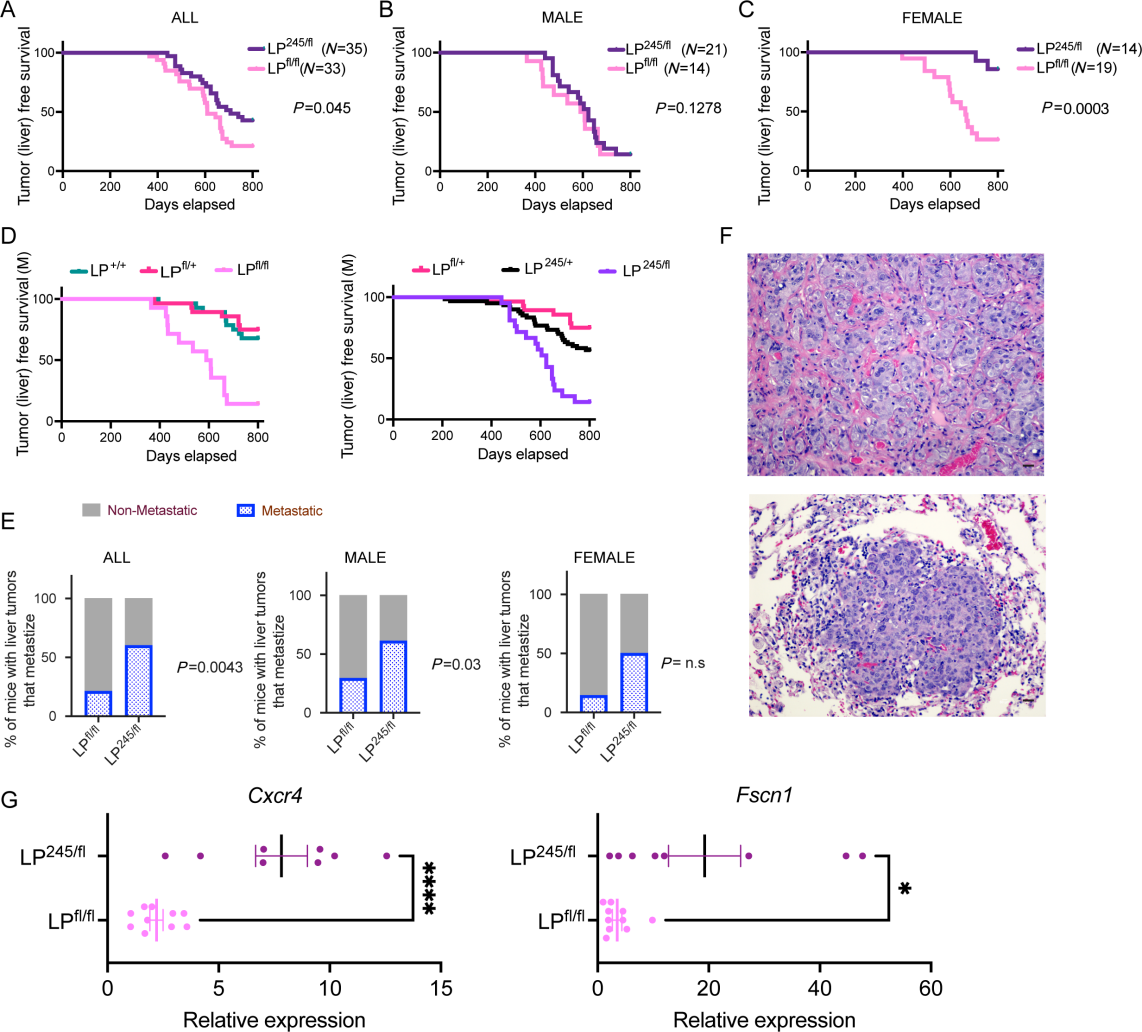
p53R245 GOF properties triple metastatic incidence in liver tumors when compared with *Trp53* loss. **A**–**C,** Kaplan–Meier survival curves for animals with genotypes LP^245/fl^ (*N* = 35), LP^fl/fl^ (*N* = 33) with hepatocellular adenomas and carcinomas in all animals (A) or split by gender (B and C). **D,** Kaplan–Meier survival curves for animals (male) with indicated genotypes, with hepatocellular adenomas and carcinomas. **E,** Comparison of metastases incidence based on indicated genotypes, in all animals (left) or split by gender (middle and right). **F,** Representative H&E-stained sections of liver metastasis in the lung. Scale bars, 100 µm. **G,** qRT-PCR analysis for *Cxcr4*, and *Fscn1*, in liver tumors from LP^245/fl^ (*N* = 8) or LP^fl/fl^ (*N* = 10) mice. Each dot is an independent liver tumor. *, *P* < 0.05; ****, *P* < 0.0001.

Next, we aged LP^245/fl^ (*N* = 35) and LP^fl/fl^ (*N* = 33) and assessed survival. LP^fl/fl^ had a shorter liver tumor-free survival than LP^245/fl^, with median survival of 609 and 707 days, respectively (Fig. 5A). However, these differences were due to gender. While no differences in liver tumor-free survival were observed among males (Fig. 5B), unexpectantly, LP^245/fl^ females had a significantly longer liver tumor-free survival than LP^fl/fl^ ones (Fig. 5C) due to lower incidence of tumors in these mice. The overall survival between these two cohorts (Supplementary Fig. S4) indicated that animals from both cohorts were monitored for similar time frames, therefore LP^245/fl^ females had ample time to develop tumors. In both cohorts, loss of the second WT allele significantly accelerated liver tumor formation, suggesting that even low levels of WT p53 are significantly protective (Fig. 5D).

One important GOF phenotype attributed to mutant p53 is metastasis (11, 18). Tumors from LP^245/fl^ were significantly more metastatic than LP^fl/fl^ tumors; metastatic incidence of liver tumors with LP^245/fl^ was triple that of LP^fl/fl^ ones (Fig. 5E). Similarly, metastatic incidence of liver tumors with LP^245/fl^ was high across both genders (Fig. 5E). These metastases were commonly observed in the lung (Fig. 5F). Previously, a panel of genes that mediate breast cancer metastasis to the lung and associated with p53R245W mutation were identified (27). We therefore examined the expression of two of these genes, *Cxcr4* and *Fscn1*, which also drive liver metastasis (41, 42). Strikingly, the expression of *Cxcr4* and *Fscn1* were significantly enriched in LP^245/fl^ HCCs when compared with LP^fl/fl^ ones (Fig. 5G). These data suggest that metastasis is a GOF phenotype and is higher in mice with mutant p53R245 as opposed to no *Trp53*.

In sum, by generating and characterizing five cohorts with different somatic alterations of p53 in hepatocytes (LP^+/+^, LP^fl/+^, LP^245/+^, LP^fl/fl^, LP^245/fl^) and feeding them a HFCD diet, we discovered that p53R245W displays both IE and GOF properties. However, although we observe inhibitory function of p53R45W, it is the p53R245W GOF properties that contribute to increased carcinoma initiation, progression, tumor plasticity, and metastasis.

## Discussion

One important observation in our study was the p53R245W increased the incidence of mixed HCC and CCA tumors in both cohorts (LP^245/+^ and LP^245/fl^) when compared with control ones, implicating p53R248W as a driver of cancer cell plasticity. Mutant p53 interacts with other transcription factors to alter transcriptomes and is associated with protein stability (9–15, 21, 39). In our study, we found via immunofluorescence that p53R245W was particularly stable in CCA; but not in HCCs. A tight connection exists between stroma stiffness, mutant p53 stability and keratin 19 expression (43, 44). Meanwhile, mechanical cues such as increased stiffness control mutant p53 stabilization through the melanovate/Rho axis (43). CCA, differently from HCCs, have reactive desmoplastic stroma containing cancer-associated fibroblasts, increasing stiffness in the tumor. Furthermore, others have found that the presence of fibrous tumor stroma is associated with keratin 19 expression for HCCs, which is a poor prognostic marker for this disease (44–46). Mechanistically, hepatocyte growth factor from the stroma-activated c-MET and the MEK–ERK1/2 pathway in hepatocytes, which upregulated keratin 19 expression in HCC cells (44). We speculate that emergence of stroma stiffness may stabilize mutant p53, hence increase GOF, and alter identity toward bile duct lineage. Interestingly, we also observed single cell rather than duct-like organization of keratin 19-positive cells within HCCs with mutant p53. In the age of precision medicine, patients with a p53R248W missense mutation in liver tumors are more likely to metastasize and develop plastic tumors with HCC-CCA features, which are harder to treat, display more aggressive behavior and poorer prognosis. Hence patients with this mutation may require more aggressive treatments.

Another significant observation in our study is that p53R245W expressed specifically in hepatocytes increases fatty liver and fibrosis in these animals. In a separate study, we also found that another missense mutant, p53R172H, in concert with immune regulator IL27 receptor deficiency results in spontaneous and sustained liver inflammation, steatosis, and fibrosis (25). More studies are needed to elucidate the mechanism how p53R245W affects fatty liver and fibrosis.

The liver is a unique organ with the remarkable ability to regenerate upon stress or cell death. Regarding liver tumorigenesis, it is well accepted that stressors that induce cell death are then followed by compensatory proliferation. Recurrent and chronic stressors induce a vicious cycle of cell death followed by compensatory proliferation mechanisms, the latter when left unchecked, drive tumorigenesis. p53 induces various forms of cell death, including apoptosis, ferroptosis, and senescence (47). Others have shown that constitutive activation of p53 conditionally in hepatocytes (via Mdm2 deletion) induces fibrosis in the liver, and HCC at 2 years (48, 49). In addition, constitutive activation of p53 in combination with Kras mutations accelerates liver tumorigenesis in a non–cell-dependent manner (48). Thus, stress-induced, p53-mediated cell death induces a microenvironment that is prone to foster tumorigenesis. The cells that dampen the WT p53 activity are the ones to progress to malignancy in this microenvironment (48). Therefore, excessive WT p53 in the liver cells promotes cell death/compensatory mechanisms that initiate this vicious cycle; however, cells that progress to malignancy attenuate p53, indicating a critical role of p53 in the liver.

Our study indicated that p53 dosage and types of p53 alterations affect liver tumorigenesis. Loss of one WT p53 allele (therefore, 50% less p53) did not accelerate liver tumorigenesis; HFCD fed LP^fl/+^ animals had similar survival and liver tumor incidence as LP^+/+^, indicating that 50% of available p53 is sufficiently protective. On the other hand, loss of both p53 alleles significantly accelerated liver tumor incidence and lowered time-to-tumor incidence. P53R245W heterozygous mice showed significant IE on *Trp53* targets, but tumor incidence and survival were not the same as complete loss of p53, indicating partial inhibition of WT p53 functions by p53R245W. In other words, even low levels of WT p53 are protective against liver tumorigenesis. In other tumor models, a neomorph p53 allele which expresses about 7% of the WT p53 protein produced significant protection in survival when compared with *Trp53^−^^/^^−^* animals (50).

In our model, males lacking p53 in hepatocytes (LP^fl/fl^) had a higher incidence of premalignant lesions than mice with p53R245W. However, only a few of these lesions progress to carcinomas. In contrast, 55% animals of males with p53R245W (LP^245/fl^) had premalignant lesions, and 45% progressed to carcinomas, indicating strong GOF mechanisms of R245W. We speculate that this GOF may lower the cell death in the liver, blocking the signals for compensatory proliferation in premalignancy, and hence lowering the incidence of premalignant lesions. However, once premalignant lesions are formed, then p53R245W-mediated GOF properties contribute to increased malignancy and tumor progression.

Females with *Trp53* loss had higher tumor incidence in the liver than those with a mutant p53, and as abovementioned, we speculate that GOF properties may lower the cell death in the tissue, blocking the need for proliferation in premalignancy, and hence lower incidence of premalignant lesions. These observations also suggest that cell death mechanisms induced in females must be relatively weaker than in males, hence lowering tumor incidence in p53R245W females. For example, due to estrogen levels, females are significantly more protected than males to undergo ferroptotic cell death (51).

The *TP53* transcription factor is involved in response to stressors such as DNA damage, oxidative stress, inflammation, oncogenic growth, and metabolic challenges. While p53-dependent DNA damage response has been well annotated, how different p53 alterations specifically in liver cells respond to metabolic challenges remain to be explored. In this study, we discovered that p53R245W displays both IE and GOF function *in vivo*. However, it is the p53R245W GOF properties that contribute to increased carcinoma initiation, progression, tumor transdifferentiation, and metastasis.

## Supplementary Material

Supplementary Figure 1 Transcriptome analyses of the liver under metabolic challenges

Supplementary Figure 2 Tumor free survival curves of animals fed a regular or HFCD diet

Supplementary Figure 3 The status of the WT p53 allele in 19 tumors with LP245/+ genotype

Supplementary Figure 4 Kaplan-Meier survival curves for animals with indicated genotypes

Supplementary Table 1 A list of genotyping and RT-qPCR primers that were used in this study

Supplementary Table 2 141 genes that had a descending slope >1 and determination correlation coefficient R2 value >0.6

Supplementary Table 3 Gene names, E-value, and motif diagrams from the MEME MAST analysis of 141 DEGs from Supplementary Table 2 examined for p53 response elements in 10KB upstream of the start site
