# Supplementary material for: p53R245W Mutation Fuels Cancer Initiation and Metastases in NASH-driven Liver Tumorigenesis
Source: Cancer Res Commun. 2023 Dec 29;3(12):2640–52. doi: 10.1158/2767-9764.CRC-23-0218 (PMC10761659; doi:10.1158/2767-9764.CRC-23-0218)
Supplement: Supplementary Figure 1 — Transcriptome analyses of the liver under metabolic challenges [file crc-23-0218-s01.pdf]

Supplementary Figure 1

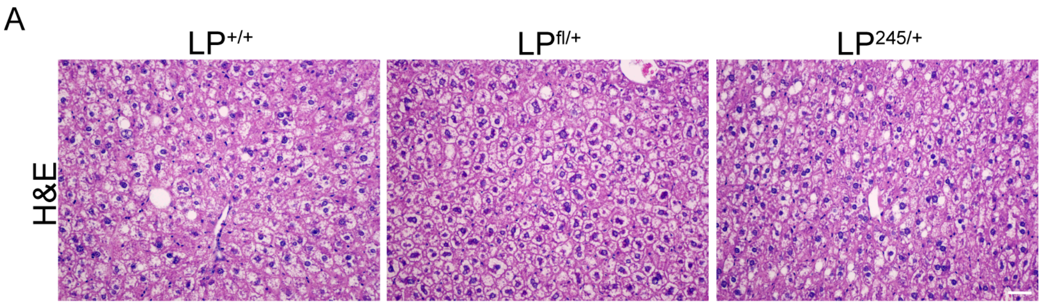

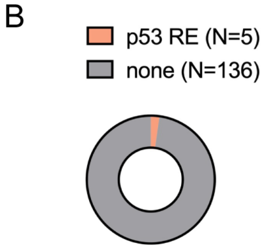

C

	P-Value	E-Value	Q-Value	TP-Value	FP value
ZNF460	3.57E-15	7.39E-12	3.34E-12	29 / 32 (90.6%)	0 / 32 (0.0%)
NR5A1	1.97E-13	4.07E-10	9.18E-11	29 / 32 (90.6%)	1 / 32 (3.1%)
ZNF281	1.03E-10	2.14E-07	3.22E-08	31 / 32 (96.9%)	6 / 32 (18.8%)

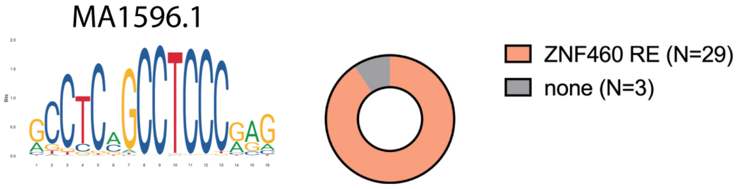

Supplementary Figure 1: Transcriptome analyses of the liver under metabolic challenges

A. Representative H&E from liver of animals from $LP^{+/+}$, $LP^{fl/+}$, and $LP^{245/+}$ after being 3 months on HFCD diet. Scale bars, 200 µm. B. Proportion of randomly selected 141 genes with p53 response element regulated by p53 using MEME MAST. C. Prediction of transcription factor upstream of the IE genes from Figure 2H using MEME SEA. TP-Value, true positive value; FP Value, false positive value.
