# Supplementary material for: p53R245W Mutation Fuels Cancer Initiation and Metastases in NASH-driven Liver Tumorigenesis
Source: Cancer Res Commun. 2023 Dec 29;3(12):2640–52. doi: 10.1158/2767-9764.CRC-23-0218 (PMC10761659; doi:10.1158/2767-9764.CRC-23-0218)
Supplement: Supplementary Figure 2 — Tumor free survival curves of animals fed a regular or HFCD diet [file crc-23-0218-s02.pdf]

Supplementary Figure 2

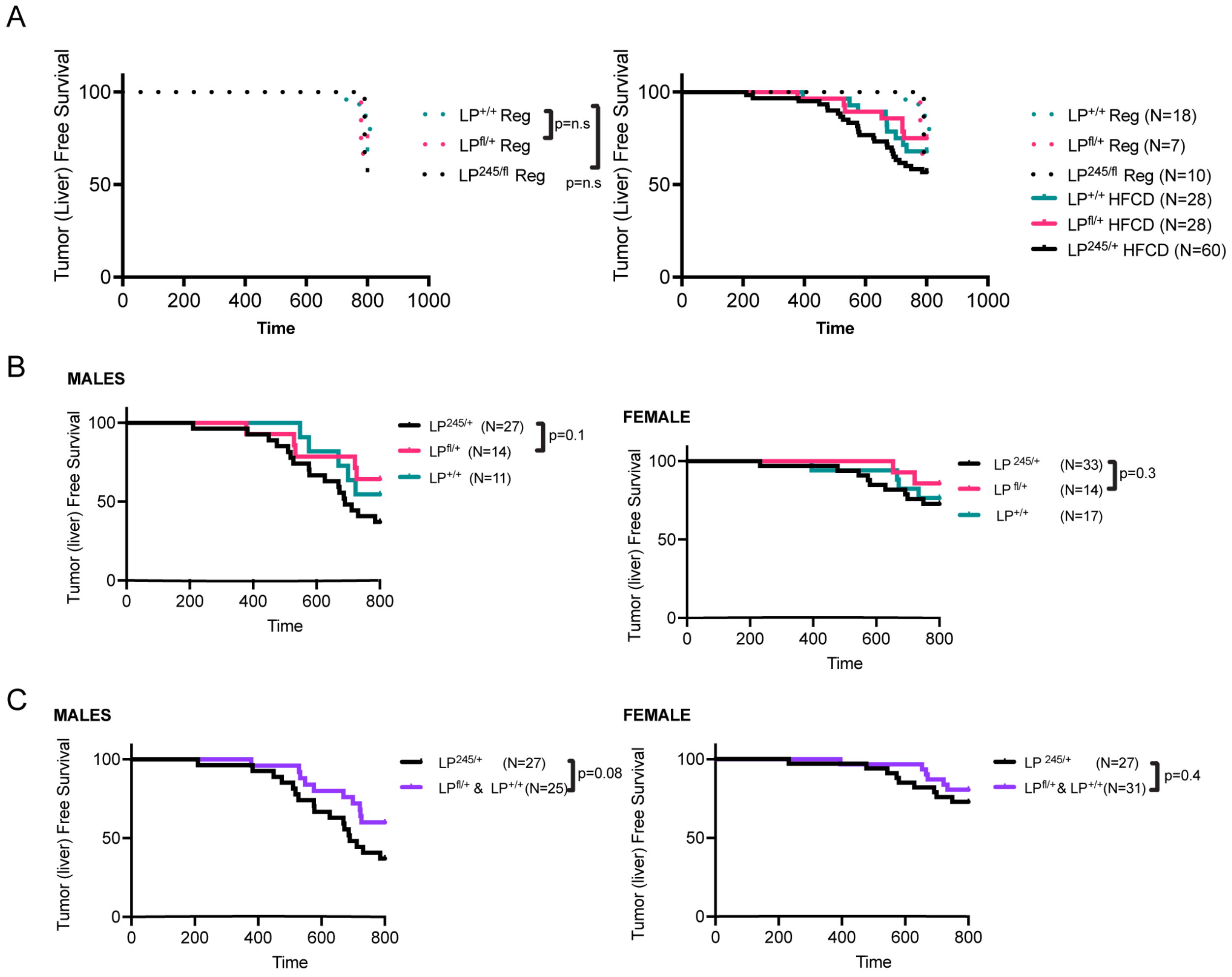

Supplementary Figure 2: Tumor free survival curves of animals fed a regular or HFCD diet. A. Kaplan-Meier tumor free survival curves for animals with indicated genotypes with hepatocellular adenomas and carcinomas in all animals fed regular diet or HFCD diet as indicated. B-C. Kaplan-Meier tumor free survival curves for animals with indicated genotypes with hepatocellular adenomas and carcinomas in all animals split by gender.
