# Supplementary material for: p53R245W Mutation Fuels Cancer Initiation and Metastases in NASH-driven Liver Tumorigenesis
Source: Cancer Res Commun. 2023 Dec 29;3(12):2640–52. doi: 10.1158/2767-9764.CRC-23-0218 (PMC10761659; doi:10.1158/2767-9764.CRC-23-0218)
Supplement: Supplementary Figure 3 — The status of the WT p53 allele in 19 tumors with LP245/+ genotype [file crc-23-0218-s03.pdf]

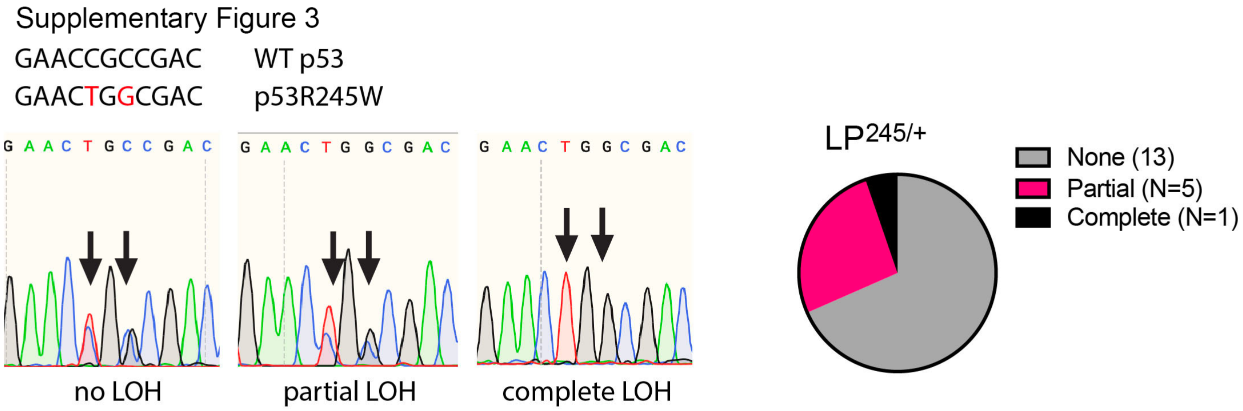

Supplementary Figure 3: The status of the WT p53 allele in 19 tumors with LP$^{245/+}$ genotype.
