# Supplementary figures and images for: p53R245W Mutation Fuels Cancer Initiation and Metastases in NASH-driven Liver Tumorigenesis
Source: Cancer Res Commun. 2023 Dec 29;3(12):2640–52. doi: 10.1158/2767-9764.CRC-23-0218 (PMC10761659; doi:10.1158/2767-9764.CRC-23-0218)

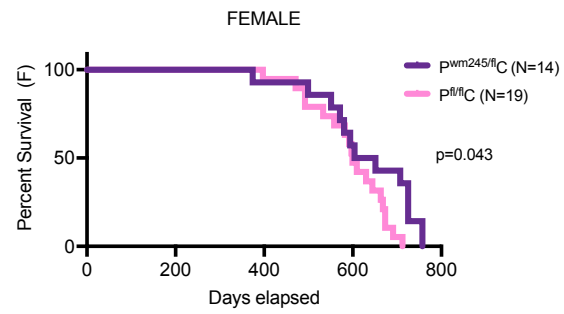

**Supplementary Figure 4: Kaplan-Meier survival curves for animals with indicated genotypes.**

Supplement: Supplementary Figure 4 — Kaplan-Meier survival curves for animals with indicated genotypes [file crc-23-0218-s04.pdf]
